# Octamer-binding transcription factor 4-positive circulating tumor cell predicts worse treatment response and survival in advanced cholangiocarcinoma patients who receive immune checkpoint inhibitors treatment

**DOI:** 10.1186/s12957-024-03369-7

**Published:** 2024-04-25

**Authors:** Fei Pei, Zhen Tao, Qi Lu, Tao Fang, Shasha Peng

**Affiliations:** Department of Hepatobiliary Pancreatic Surgery, Hubei Key Laboratory of Kidney Disease Pathogenesis and Intervention, Huangshi Central Hospital, Affiliated Hospital of Hubei Polytechnic University, No. 141 Tianjin Road, Huangshi, 435200 Hubei China

**Keywords:** Cholangiocarcinoma, Octamer-binding transcription factor 4, Circulating tumor cell, Immune checkpoint inhibitors, Prognosis

## Abstract

**Background:**

Octamer-binding transcription factor 4-positive circulating tumor cell (OCT4^+^CTC) exhibits high stemness and invasive potential, which may influence the efficacy of immune checkpoint inhibitors (ICI). This study aimed to assess the prognostic role of OCT4^+^CTC in advanced cholangiocarcinoma (CCA) patients who received ICI treatment.

**Methods:**

In total, 40 advanced CCA patients who received ICI treatment were included, and CTC and OCT4 counts were detected via a Canpatrol system and an RNA in situ hybridization method before ICI treatment. Patients were subsequently divided into none CTC, OCT4^−^CTC, and OCT4^+^CTC groups. Patients were followed up for a median of 10.4 months.

**Results:**

The percentages of patients in none CTC, OCT4^−^CTC, and OCT4^+^CTC groups were 25.0%, 30.0%, and 45.0%, respectively. The proportion of patients with lymph node metastasis was highest in OCT4^+^CTC group, followed by none CTC group, and lowest in OCT4^−^CTC group (*P* = 0.025). The objective response rate (ORR) was lowest in OCT4^+^CTC group, moderate in OCT4^−^CTC group, and highest in none CTC group (*P* = 0.009), while disease control rate was not different among three groups (*P* = 0.293). In addition, progression-free survival (PFS) (*P* < 0.001) and overall survival (OS) (*P* = 0.001) were shorter in the OCT4^+^CTC group than in none CTC & OCT4^−^CTC group. Moreover, OCT4^+^CTC (versus none CTC) was independently linked with poorer PFS [hazard ratio (HR) = 6.752, *P* = 0.001] and OS (HR = 6.674, *P* = 0.003) in advanced CCA patients.

**Conclusion:**

OCT4^+^CTC relates to lymph node metastasis and shows a good predictive value for poor treatment response and survival in advanced CCA patients who receive ICI treatment.

**Supplementary Information:**

The online version contains supplementary material available at 10.1186/s12957-024-03369-7.

## Introduction

Cholangiocarcinoma (CCA) is a highly fatal malignancy of the hepatobiliary system that can be classified as intrahepatic, perihilar, or distal CCA [1, 2]. Because there are no clinical symptoms in the early stage of CCA, most CCA patients are diagnosed at an advanced stage [3, 4]. Unfortunately, although the systemic treatment scenarios for advanced CCA patients are continuously explored, the survival advantage of front-line therapy is only modest and the second-line treatment remains controversial in the scientific community [5–10]. In recent years, immunotherapy has gradually become one of the most important therapeutic strategies for advanced CCA patients [11, 12]. Especially, immune checkpoint inhibitors (ICI) treatment is deemed to enhance antitumor immune response and thus improve patients’ survival outcomes [13–15]. However, not all advanced CCA patients respond well to ICI treatment [16, 17]. Thus, predicting the efficacy of ICI treatment in advanced CCA patients is a noteworthy issue.

Cancer stemness promotes tumorigenesis, invasion, and metastasis, and enhances resistance to cancer treatment, which affects the treatment efficacy of ICI treatment [18–21]. Notably, the stem cell marker octamer-binding transcription factor 4 (OCT4) maintains the pluripotency and self-renewal potential of stem cells and is reported to induce drug resistance in CCA cell lines [22–24]. Circulating tumor cell (CTC) is one type of tumor cells that sheds from solid tumor lesions and enters the blood circulation, which represents the high invasiveness of tumor cells [25]. Notably, the CTC constitutes a subset of cells that exhibit cancer stemness characteristics [25–27]. Previous studies suggest that CTC with stem cell-like properties is related to tumor metastasis and relapse in some cancer patients, such as those with colon cancer, gastric cancer, and hepatocellular carcinoma [28–30]. The above findings indicate that OCT4^+^CTC not only reflects resistance to cancer therapies, but also represents tumor invasion and metastasis. Thus, it is reasonable to hypothesize that OCT4^+^CTC can reflect the response to ICI treatment in advanced CCA patients. However, there is a lack of relevant related research.

Therefore, this study aimed to evaluate the ability of OCT4^+^CTC to predict treatment response and survival in advanced CCA patients who received ICI treatment.

## Materials and methods

### Population

A total of 40 advanced CCA patients who received ICI treatment between February 2020 and February 2023 were enrolled in this study. The inclusion criteria were as follows: (i) histopathologically confirmed advanced CCA; (ii) 18 years old or older; (iii) about to receive ICI treatment; (iv) had measurable lesions for evaluation; (v) had an Eastern Cooperative Oncology Group performance status (ECOG PS) ≤ 2; and (vi) were willing to cooperate with this study. The exclusion criteria were as follows: (i) no adequate bone marrow, hepatic, or kidney function; (ii) expected survival time of fewer than 3 months; and (iii) pregnancy or lactation. All patients provided informed consent. The Ethics Committee approved this study.

### Data collection and ICI treatment

Demographics, disease characteristics, and information on previous treatments were collected from advanced CCA patients. In this study, patients received ICI treatment until disease progression or severe toxicity occurred. ICI medicine used included camrelizumab, sintilimab, pembrolizumab, nivolumab, and durvalumab. In addition, combination treatment was not limited, which included capecitabine + oxaliplatin, lenvatinib, gemcitabine + cisplatin, and gemcitabine + S-1. The specific doses of medicine used were selected according to patient disease status and according to the Chinese Society of Clinical Oncology (CSCO) guidelines [31].

### CTC information

Before ICI treatment initiation, peripheral blood samples were collected to determine the CTC count using a Canpatrol system [32]. A sample was labeled as ‘CTC’ when at least one CTC was detected per 5 mL of peripheral blood; otherwise, the sample was labeled as ‘None CTC’. Furthermore, OCT4 expression in CTC was detected via an RNA in situ hybridization method [33]. If more than or equal to 1 CTC exhibited OCT4, the sample was labeled as ‘OCT4^+^CTC’; while if no OCT4 was observed in CTC, the sample was labeled as ‘OCT4^−^CTC’.

### Assessment

Patients were routinely followed up with a median follow-up time of 10.4 months and a range from 1.3 to 26.1 months. The last follow-up date was April 2023. During the follow-up, imaging examinations were conducted once every two cycles (approximately 1.4 months) for the first 6 months, every 2 months for the next 6 months, and every 3 months thereafter. Based on the results of imaging examinations after 2 cycles of treatment initiation, treatment responses were assessed via Response Evaluation Criteria in Solid Tumors (RECIST) v1.1. Additionally, progression-free survival (PFS) and overall survival (OS) were calculated.

### Statistical analysis

SPSS v. 26.0 (IBM, USA) was used for the data analysis. χ^2^ test and Fisher’s exact test were used for comparison analyses. Kaplan-Meier curves were used to evaluate PFS and OS, and the Log-rank test was used. The factors related to PFS or OS were determined via Cox regression analyses, in which the backward stepwise method was used in the multivariate analyses. *P* < 0.05 indicated statistical significance.

## Results

### Baseline features of advanced CCA patients

The enrolled advanced CCA patients included 13 (32.5%) females and 27 (67.5%) males, whose mean age was 62.2 ± 9.4 years. There were 22 (55.0%) patients with intrahepatic lesions, 14 (35.0%) patients with hilar lesions, and 4 (10.0%) patients with extrahepatic lesions. Notably, 27 (67.5%) patients had lymph node metastasis and 27 (67.5%) patients had distant metastasis. Among the 40 patients, 12 (30.0%) patients had tumor-node-metastasis (TNM) stage III disease, and 28 (70.0%) patients had TNM stage IV disease. In addition, other clinical characteristics of the patients were displayed in Table 1.

**Table 1:** Clinical characteristics of advanced CCA patients

Characteristics	CCA patients (*N* = 40)
Age (years), mean ± SD	62.2 ± 9.4
Sex, n (%)	
Female	13 (32.5)
Male	27 (67.5)
ECOG PS score, n (%)	
0	4 (10.0)
1	26 (65.0)
2	10 (25.0)
Lesion location, n (%)	
Intrahepatic	22 (55.0)
Hilar	14 (35.0)
Extrahepatic	4 (10.0)
Lymph node metastasis, n (%)	27 (67.5)
Distant metastasis, n (%)	27 (67.5)
TNM stage, n (%)	
III	12 (30.0)
IV	28 (70.0)
Abnormal CA199, n (%)	33 (82.5)
Abnormal CA125, n (%)	25 (62.5)
Abnormal CEA, n (%)	16 (40.0)
Previous surgery, n (%)	9 (22.5)
Previous chemotherapy, n (%)	19 (47.5)
Previous radiotherapy, n (%)	5 (12.5)

CCA: cholangiocarcinoma; SD: standard deviation; ECOG PS: Eastern Cooperative Oncology Group performance status; TNM: tumor-node-metastasis; CA199: cancer antigen 19 − 9; CA125: cancer antigen 125; CEA: carcinoembryonic antigen

### Treatment regimens for advanced CCA patients

There were 21 (52.5%) patients who received first-line treatment and 19 (47.5%) patients who underwent second or above-line treatment at enrollment. All patients received ICI treatment and combination treatment. Regarding the ICI regimen, 19 (47.5%) patients took camrelizumab, 9 (22.5%) patients received sintilimab, 7 (17.5%) patients received pembrolizumab, 3 (7.5%) patients were treated with nivolumab, and 2 (5.0%) patients received durvalumab. Moreover, all patients received combination treatment. Specifically, 15 (37.5%) patients received capecitabine + oxaliplatin, 10 (25.0%) patients took lenvatinib, 8 (20.0%) patients received gemcitabine + cisplatin, and 7 (17.5%) patients were treated with gemcitabine + S-1 (Table 2).

**Table 2:** Treatment regimens

Items	CCA patients (*N* = 40)
Treatment line, n (%)	
First	21 (52.5)
Second or above	19 (47.5)
ICI treatment, n (%)	40 (100.0)
ICI regimen, n (%)	
Camrelizumab	19 (47.5)
Sintilimab	9 (22.5)
Pembrolizumab	7 (17.5)
Nivolumab	3 (7.5)
Durvalumab	2 (5.0)
Combination treatment, n (%)	40 (100.0)
Combination treatment regimen, n (%)	
Capecitabine + oxaliplatin	15 (37.5)
Lenvatinib	10 (25.0)
Gemcitabine + cisplatin	8 (20.0)
Gemcitabine + S-1	7 (17.5)

CCA: cholangiocarcinoma; ICI: immune checkpoint inhibitor

### Comparison of baseline features and treatment regimens among groups of advanced CCA patients

**Fig. 1:**
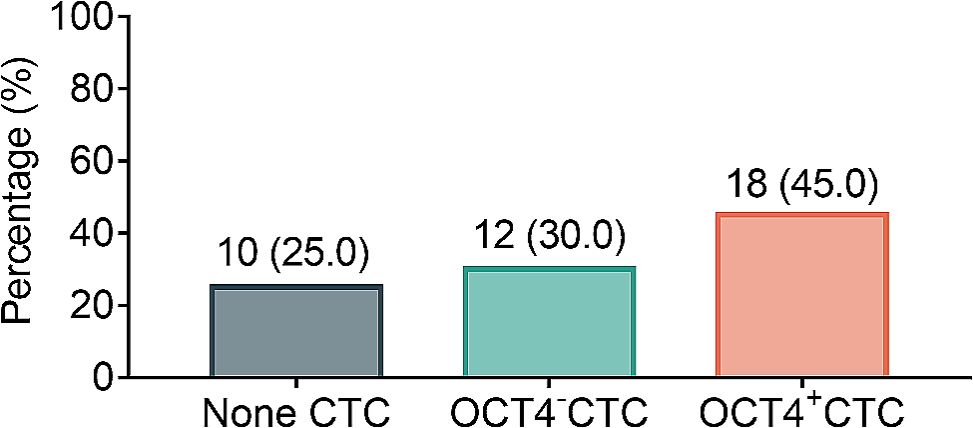
The proportions of advanced CCA patients with none CTC, OCT4^−^CTC, and OCT4^+^CTC

The percentages of patients in none CTC, OCT4^−^CTC, and OCT4^+^CTC groups were 25.0%, 30.0%, and 45.0%, respectively (Fig. 1). Interestingly, the proportion of patients with lymph node metastasis was highest in the OCT4^+^CTC group, moderate in the none CTC group, and lowest in the OCT4^−^CTC group (*P* = 0.025). The proportion of patients with distant metastasis was different among the three groups, but the difference was not significant (*P* = 0.065). Furthermore, no difference was observed in other clinical characteristics or in the use of any treatment regimens among the three groups (all *P* > 0.05) (Table 3).

**Table 3:** Comparison analyses of clinical characteristics and treatment regimens

Characteristics	None CTC (*n* = 10)	OCT4^−^CTC (*n* = 12)	OCT4^+^CTC (*n* = 18)	*P* value
Age, n (%)				0.118
< 65 years	3 (30.0)	9 (75.0)	10 (55.6)	
≥ 65 years	7 (70.0)	3 (25.0)	8 (44.4)	
Sex, n (%)				0.567
Female	2 (20.0)	5 (41.7)	6 (33.3)	
Male	8 (80.0)	7 (58.3)	12 (66.7)	
ECOG PS score, n (%)				0.175
0	3 (30.0)	1 (8.3)	0 (0.0)	
1	5 (50.0)	8 (66.7)	13 (72.2)	
2	2 (20.0)	3 (25.0)	5 (27.8)	
Lesion location, n (%)				0.666
Intrahepatic	6 (60.0)	5 (41.7)	11 (61.1)	
Hilar	4 (40.0)	5 (41.7)	5 (27.8)	
Extrahepatic	0 (0.0)	2 (16.7)	2 (11.1)	
Lymph node metastasis, n (%)	6 (60.0)	5 (41.7)	16 (88.9)	0.025
Distant metastasis, n (%)	4 (40.0)	8 (66.7)	15 (83.3)	0.065
TNM stage, n (%)				0.184
III	5 (50.0)	4 (33.3)	3 (16.7)	
IV	5 (50.0)	8 (66.7)	15 (83.3)	
Abnormal CA199, n (%)	7 (70.0)	9 (75.0)	17 (94.4)	0.205
Abnormal CA125, n (%)	4 (40.0)	7 (58.3)	14 (77.8)	0.151
Abnormal CEA, n (%)	3 (30.0)	3 (25.0)	10 (55.6)	0.206
Previous surgery, n (%)	4 (40.0)	1 (8.3)	4 (22.2)	0.237
Previous chemotherapy, n (%)	2 (20.0)	5 (41.7)	12 (66.7)	0.055
Previous radiotherapy, n (%)	1 (10.0)	1 (8.3)	3 (16.7)	0.847
Treatment line, n (%)				0.055
First	8 (80.0)	7 (58.3)	6 (33.3)	
Second or above	2 (20.0)	5 (41.7)	12 (66.7)	
ICI treatment, n (%)	10 (100.0)	12 (100.0)	18 (100.0)	(-)
ICI regimen, n (%)				0.876
Camrelizumab	5 (50.0)	6 (50.0)	8 (44.4)	
Sintilimab	2 (20.0)	2 (16.7)	5 (27.8)	
Pembrolizumab	1 (10.0)	3 (25.0)	3 (16.7)	
Nivolumab	2 (20.0)	0 (0.0)	1 (5.6)	
Durvalumab	0 (0.0)	1 (8.3)	1 (5.6)	
Combination treatment, n (%)	10 (100.0)	12 (100.0)	18 (100.0)	(-)
Combination treatment regimen, n (%)				0.174
Capecitabine + oxaliplatin	3 (30.0)	6 (50.0)	6 (33.3)	
Lenvatinib	1 (10.0)	1 (8.3)	8 (44.4)	
Gemcitabine + cisplatin	3 (30.0)	2 (16.7)	3 (16.7)	
Gemcitabine + S-1	3 (30.0)	3 (25.0)	1 (5.6)	

CTC: circulating tumor cell; OCT4: octamer-binding transcription factor 4; ECOG PS: Eastern Cooperative Oncology Group performance status; TNM: tumor-node-metastasis; CA199: cancer antigen 19 − 9; CA125: cancer antigen 125; CEA: carcinoembryonic antigen; ICI: immune checkpoint inhibitor

### Comparison of treatment response among groups of advanced CCA patients

The objective response rates (ORRs) were 80.0%, 58.3%, and 22.2% in the none CTC, OCT4^−^CTC, and OCT4^+^CTC groups, respectively. The ORR was lowest in the OCT4^+^CTC group, followed by the OCT4^−^CTC group, and highest in the none CTC group (*P* = 0.009). In addition, the disease control rates (DCRs) were 100%, 91.7%, and 77.8% in the none CTC, OCT4^−^CTC, and OCT4^+^CTC groups, respectively. There was no discrepancy in the DCR among the three groups (*P* = 0.293) (Table 4). In addition, the influence of lesion location on ICI treatment effectiveness was explored in advanced CCA patients, which showed that there was no difference in ORR (*P* = 0.960) or DCR (*P* = 0.452) among advanced CCA patients with different lesion location Supplementary Table 1).

**Table 4:** Comparison analyses of treatment response

Items	None CTC (*n* = 10)	OCT4^−^CTC (*n* = 12)	OCT4^+^CTC (*n* = 18)	*P* value
Treatment response, n (%)				(-)
CR	0 (0.0)	0 (0.0)	0 (0.0)	
PR	8 (80.0)	7 (58.3)	4 (22.2)	
SD	2 (20.0)	4 (33.4)	10 (55.6)	
PD	0 (0.0)	1 (8.3)	4 (22.2)	
ORR, n (%)	8 (80.0)	7 (58.3)	4 (22.2)	0.009
DCR, n (%)	10 (100.0)	11 (91.7)	14 (77.8)	0.293

CTC: circulating tumor cell; OCT4: octamer-binding transcription factor 4; CR: complete response; PR: partial response; SD: stable disease; PD: progressive disease; ORR: objective response rate; DCR: disease control rate

### Comparison of PFS and OS among groups of advanced CCA patients

**Fig. 2:**
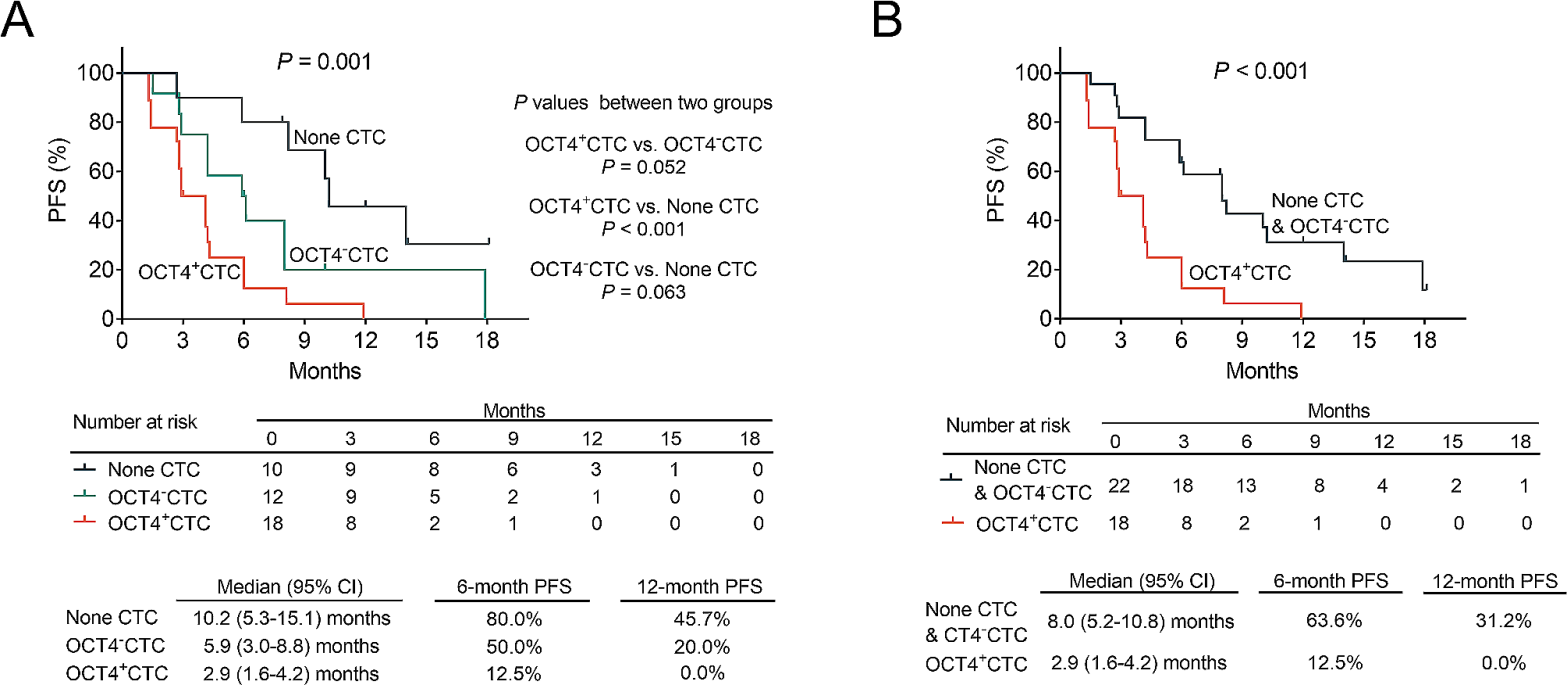
PFS in the none CTC, OCT4^−^CTC, and OCT4^+^CTC groups. Comparison of PFS among none CTC, OCT4^−^CTC, and OCT4^+^CTC groups (**A**); comparison of PFS between none CTC & OCT4^−^CTC and OCT4^+^CTC groups (**B**)

The 6-month PFS rates were 80.0%, 50.0%, and 12.5%, and the 12-month PFS rates were 45.7%, 20.0%, and 0.0% in the none CTC, OCT4^−^CTC, and OCT4^+^CTC groups, respectively. Moreover, PFS was shortest in the OCT4^+^CTC group, medium in the OCT4^−^CTC group, and longest in the none CTC group (*P* = 0.001). Pairwise comparative analyses between groups showed that the PFS was lower in the OCT4^+^CTC group than in the none CTC group (*P* < 0.001); moreover, PFS was not different between the OCT4^+^CTC group and the OCT4^−^CTC group (*P* = 0.052) or between the OCT4^−^CTC group and the none CTC group (*P* = 0.063) (Fig. 2A). The 6-month PFS rates were 63.6% and 12.5%, and the 12-month PFS rates were 31.2% and 0.0% in the none CTC & OCT4^−^CTC group and OCT4^+^CTC groups, respectively. Further comparative analysis of PFS showed that PFS was shorter in the OCT4^+^CTC group than in none CTC & OCT4^−^CTC group (*P* < 0.001) (Fig. 2B).

**Fig. 3:**
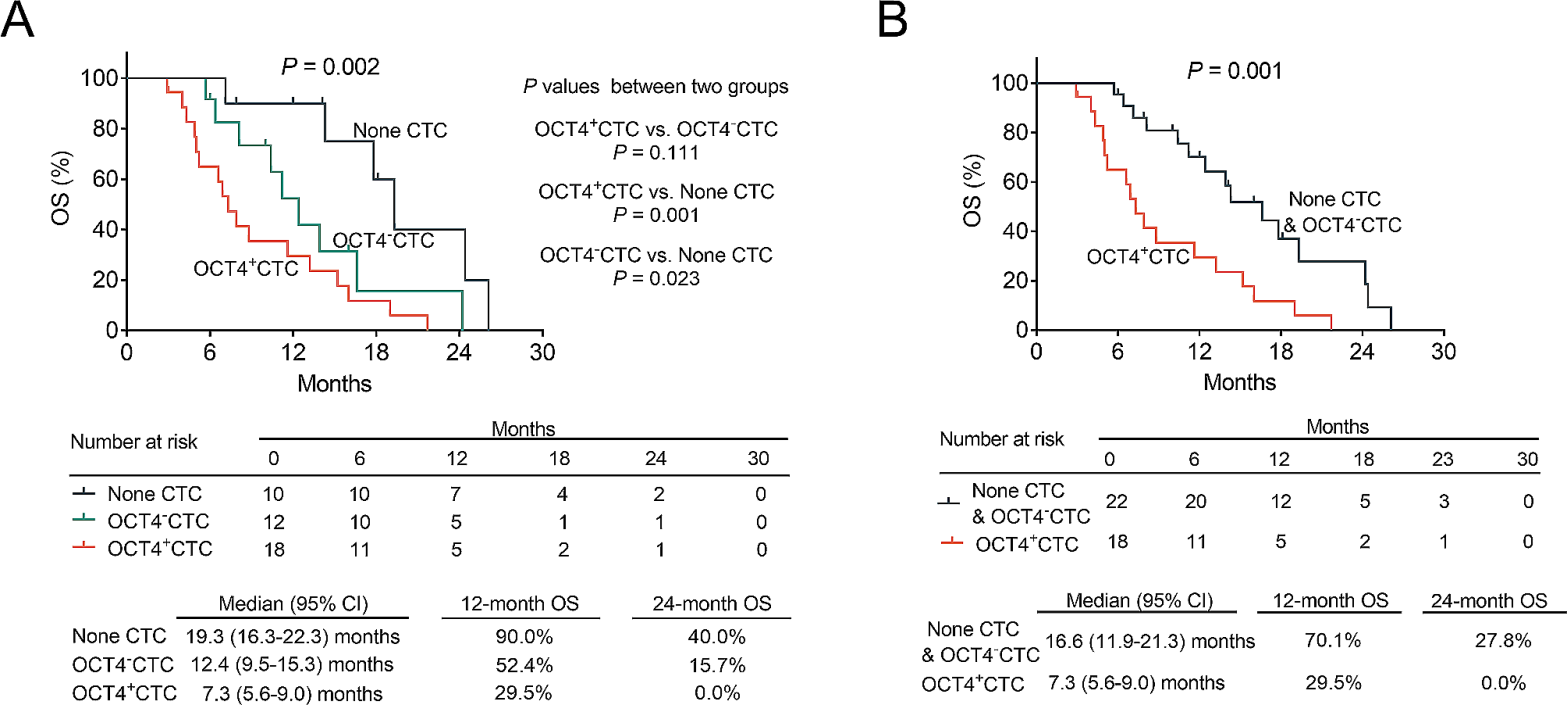
OS in the none CTC, OCT4^−^CTC, and OCT4^+^CTC groups. Comparison of OS among none CTC, OCT4^−^CTC, and OCT4^+^CTC groups (**A**); comparison of OS between none CTC & OCT4^−^CTC and OCT4^+^CTC groups (**B**)

The 12-month OS rates were 90.0%, 52.4%, and 29.5%, and the 24-month OS rates were 40.0%, 15.7%, and 0.0% in the none CTC, OCT4^−^CTC, and OCT4^+^CTC groups, respectively. OS differed among the three groups, which suggested that OS was shortest in the OCT4^+^CTC group, followed by the OCT4^−^CTC group, and longest in the none CTC group (*P* = 0.002). The pairwise comparative analyses between groups suggested that OS was shorter in the OCT4^+^CTC group than in the none CTC group (*P* = 0.001) and in the OCT4^−^CTC group than in the none CTC group (*P* = 0.023); moreover, no discrepancy was found between the OCT4^+^CTC group and the OCT4^−^CTC group (*P* = 0.111) (Fig. 3A). Additionally, the 12-month OS rates were 70.1% and 29.5%, and the 24-month OS rates were 27.8% and 0.0% in the none CTC & OCT4^−^CTC group and OCT4^+^CTC groups. A further comparison analysis of OS exhibited that OS was poorer in the OCT4^+^CTC group than in none CTC & OCT4^−^CTC group (*P* = 0.001) (Fig. 3B).

### Factors correlated with PFS and OS in advanced CCA patients

**Fig. 4:**
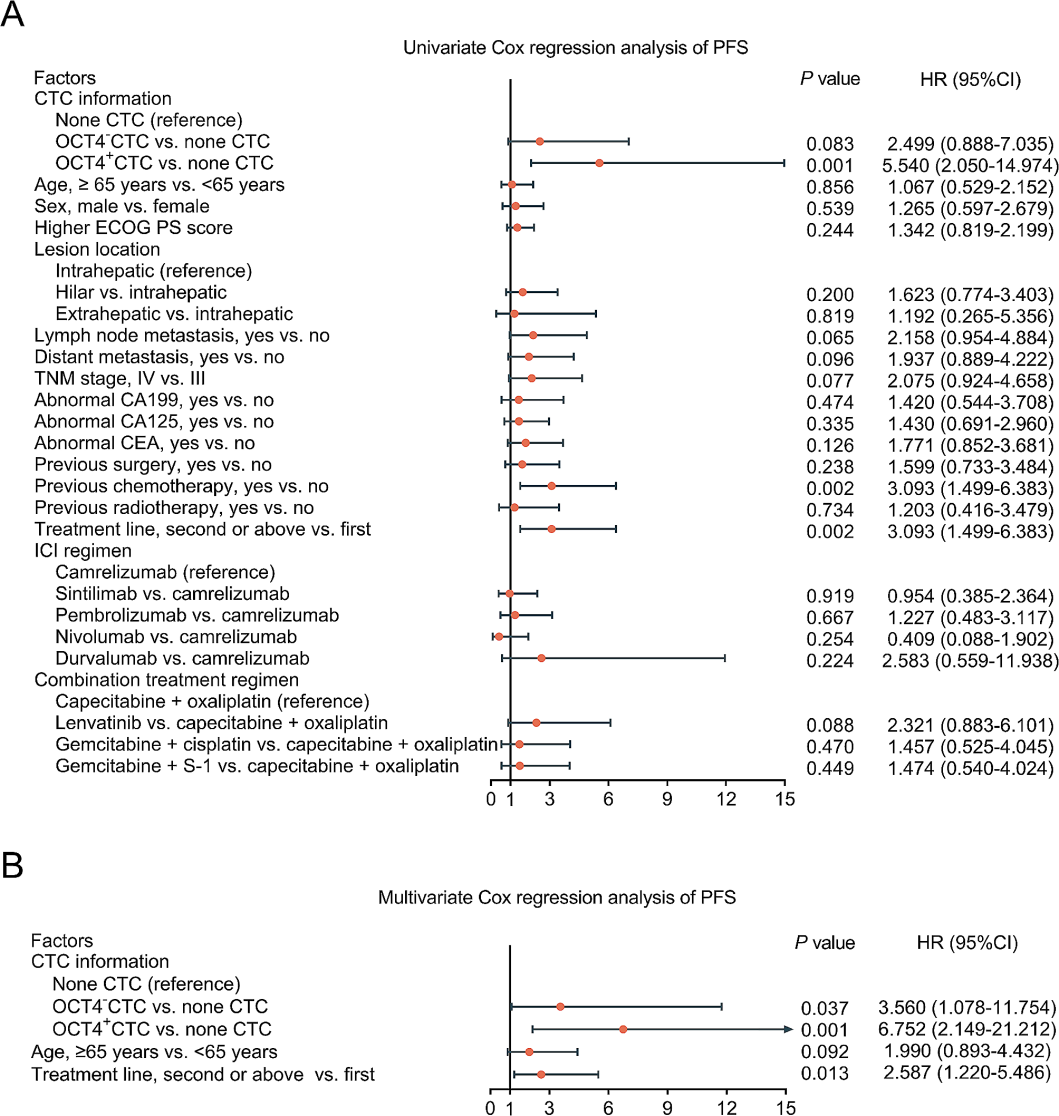
Univariate and multivariate Cox regression analyses of PFS in advanced CCA patients. Univariate Cox regression analysis was used to predict PFS (**A**); multivariate Cox regression analysis was used to identify independent predictors of PFS (**B**) in advanced CCA patients

Based on univariate Cox regression analysis, OCT4^+^CTC (versus none CTC) [hazard ratio (HR) = 5.540, *P* = 0.001], previous chemotherapy (versus no) (HR = 3.093, *P* = 0.002), and treatment line of second or above (versus first) (HR = 3.093, *P* = 0.002) were related to shorter PFS in advanced CCA patients (Fig. 4A). Moreover, the multivariate Cox regression analysis revealed that OCT4^−^CTC (versus none CTC) (HR = 3.560, *P* = 0.037), OCT4^+^CTC (versus none CTC) (HR = 6.752, *P* = 0.001), and treatment line of second or above (versus first) (HR = 2.587, *P* = 0.013) were independently associated with poorer PFS in advanced CCA patients (Fig. 4B).

**Fig. 5:**
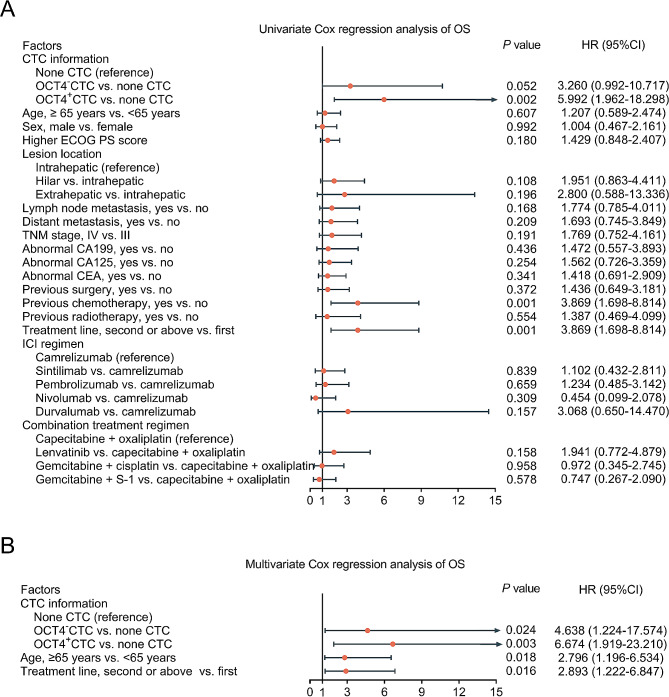
Univariate and multivariate Cox regression analyses of OS in advanced CCA patients. Univariate Cox regression analysis was used to predict OS (**A**); multivariate Cox regression analysis was used to identify independent predictors of OS (**B**) in advanced CCA patients

Moreover, OCT4^+^CTC (versus none CTC) (HR = 5.992, *P* = 0.002), previous chemotherapy (no) (HR = 3.869, *P* = 0.001), and treatment line of second or above (versus first) (HR = 3.869, *P* = 0.001) were correlated with poorer OS in advanced CCA patients (Fig. 5A). The multivariate Cox regression analysis revealed that OCT4^−^CTC (versus none CTC) (HR = 4.638, *P* = 0.024), OCT4^+^CTC (versus none CTC) (HR = 6.674, *P* = 0.003), age ≥ 65 years (versus < 65 years) (HR = 2.796, *P* = 0.018) and treatment line of second or above (versus first) (HR = 2.893, *P* = 0.016) were independently linked with shorter OS in advanced CCA patients (Fig. 5B).

## Discussion

The incidence of CCA is relatively low (0.3–85 cases per 100,000 people), and even fewer advanced CCA patients receive ICI treatment [34, 35]. Our study included as many advanced CCA patients who received ICI treatment as possible, for a total of 40 patients. The 40 patients were divided into none CTC, OCT4^−^CTC, and OCT4^+^CTC groups, with proportions of 25.0%, 30.0%, and 45.0%, respectively. Subsequent comparisons of clinical characteristics among the three groups revealed that the proportion of patients with lymph node metastasis was the highest in the OCT4^+^CTC group, followed by the none CTC group, and was the lowest in the OCT4^−^CTC group. The possible explanations were as follows: (1) OCT4^+^ indicated that tumor cells had high stemness, thus promoting tumor cell metastasis to the lymph node [36]. (2) OCT4 activated the lymphoid enhancer binding factor 1/β-catenin dependent WNT signaling pathway to induce epithelial-mesenchymal transition (EMT), thereby enhancing tumor cell metastasis [37]. (3) The presence of CTC indicated high invasiveness of tumor cells; thus, tumor cells were more likely to invade lymph nodes [38]. (4) CTC might cause lymph node metastasis through EMT [39]. Therefore, the proportion of advanced CCA patients who received ICI treatment with lymph node metastasis was the highest in the OCT4^+^CTC group.

ICI treatment boosts the host immune system, attacks tumors, and inhibits tumor growth, and is currently a new option for treating many malignant tumors, including CCA [40]. However, not all advanced CCA patients respond well to ICI treatment, thus predicting the efficacy of ICI treatment in advanced CCA patients is important [16, 17]. Interestingly, there is a general negative correlation between cancer stemness and anticancer immunity, thus cancer stemness might affect the efficacy of ICI treatment in cancer patients [41, 42]. OCT4^+^CTC represented highly stemmed tumor cells, and may be suitable for evaluating the efficacy of ICI treatment in advanced CCA patients [25, 36]. However, there are no relevant studies on this topic. Our study suggested that the ORR was lowest in the OCT4^+^CTC group, modest in the OCT4^−^CTC group, and highest in the none CTC group. This finding might be attributed to the following reasons: (1) OCT4 might promote the WNT/β-catenin signal pathway [43]; meanwhile, the WNT/β-catenin signaling pathway inhibited antitumor immunity [44]. (2) CTC escaped immune surveillance in the blood, which might reduce the efficacy of ICI treatment [25]. (3) OCT4^+^CTC might promote immune escape through EMT [39, 45, 46]. Thus, OCT4^+^CTC predicted poor ICI treatment response in advanced CCA patients. Notably, personalized immunotherapy strategies based on patient-specific tumor characteristics have been considered to be a necessary issue for advanced CCA patients [47, 48]. The results of our study illustrated that OCT4^+^CTC could predict treatment response in advanced CCA patients who received ICI treatment, which was helpful for stratified management and personalized treatment of these patients.

One previous study has revealed that OCT4^+^CTC has good prognostic value in patients with non-small-cell lung cancer [33]. However, the efficacy of OCT4^+^CTC in predicting the survival of advanced CCA patients who receive ICI treatment has not been determined. Our study found that both PFS and OS were lower in the OCT4^+^CTC group than in none CTC & OCT4^−^CTC group. Meanwhile, OCT4^+^CTC was independently correlated with shorter PFS and OS in advanced CCA patients who received ICI treatment. The probable reasons were as follows: (1) OCT4^+^CTC promoted tumor invasion and therapeutic resistance through EMT, which might be associated with poorer PFS and OS [39, 45, 46]. (2) OCT4^+^CTC exhibited high cancer stemness, contributing to tumor progression and recurrence; thus, the PFS and OS were reduced in the OCT4^+^CTC group [25, 49]. (3) As mentioned above, OCT4^+^CTC was related to poor ICI treatment response, which might lead to shorter PFS and OS. Therefore, OCT4^+^CTC exhibited a good prognostic value in advanced CCA patients who received ICI treatment. Overall, our study assessed the ability of OCT4^+^CTC to predict treatment response and survival in advanced CCA patients who received ICI treatment, which showed that OCT4^+^CTC had potential prognostic values in these patients. The findings of our study might further contribute to the clinical management of these patients.

There were several limitations in our study: (1) The incidence of CCA is relatively low, and the number of advanced CCA patients who receive ICI treatment is even fewer [34, 35]. Therefore, although our study included as many patients as possible, the sample size was still relatively small (*N* = 40), which might affect the generalizability of the results. (2) Our study was single-center, which limited the external validity of the study. (3) The molecular mechanism of OCT4^+^CTC linked with treatment outcomes should be investigated.

It is hypothesized that in the next five years, more and more studies will focus on the ability of OCT4^+^CTC or biomarkers to predict treatment response and survival in cancer patients who receive ICI treatment. Large-scale and multi-center studies are necessary in the future. Moreover, further studies also should explore the molecular mechanism of OCT4^+^CTC related to treatment outcomes. Notably, metformin and microbiota are thought to play important roles in the pathogenesis and progression of CCA [50, 51]. Thus, exploring the mechanisms linking OCT4^+^CTC to metformin or microbiota may also be a promising research direction.

## Conclusions

In summary, OCT4^+^CTC is associated with undesirable ORR, PFS, and OS, and has a potential prognostic value in advanced CCA patients who receive ICI treatment.

### Electronic supplementary material

Below is the link to the electronic supplementary material.

Supplementary Material 1

## Data Availability

The datasets used and/or analysed during the current study are available from the corresponding author on reasonable request.
