# Supplementary material for: Octamer-binding transcription factor 4-positive circulating tumor cell predicts worse treatment response and survival in advanced cholangiocarcinoma patients who receive immune checkpoint inhibitors treatment
Source: World J Surg Oncol. 2024 Apr 25;22:110. doi: 10.1186/s12957-024-03369-7 (PMC11044354; doi:10.1186/s12957-024-03369-7)
Supplement: Supplementary file 1 — Supplementary Material 1 [file 12957_2024_3369_MOESM1_ESM.docx]

**Supplementary Table 1.** The influence of lesion location on ICI treatment effectiveness.

| Items | Lesion location | | | | *P* value |
| --- | --- | --- | --- | --- | --- |
|  | Intrahepatic  (n = 22) | | Hilar  (n = 14) | Extrahepatic  (n = 4) |  |
| Treatment response, n (%) | |  |  |  | (-) |
| CR | 0 (0.0) | | 0 (0.0) | 0 (0.0) |  |
| PR | 10 (45.5) | | 7 (50.0) | 2 (50.0) |  |
| SD | 8 (36.4) | | 6 (42.9) | 2 (50.0) |  |
| PD | 4 (18.2) | | 1 (7.1) | 0 (0.0) |  |
| ORR, n (%) | 10 (45.4) | | 7 (50.0) | 2 (50.0) | 0.960 |
| DCR, n (%) | 18 (81.8) | | 13 (92.9) | 4 (100.0) | 0.452 |

ICI, immune checkpoint inhibitors; CR, complete response; PR, partial response; SD, stable disease; PD, progressive disease; ORR, objective response rate; DCR, disease control rate.
